# To Vaccinate or Not to Vaccinate

**DOI:** 10.1155/S1064744994000566

**Published:** 1994

**Authors:** Sebastian Faro


					Infectious Diseases in Obstetrics and Gynecology 2:153 (1994)
(C) 1994 Wiley-Liss, Inc.
To Vaccinate or Not to Vaccinate
Hepatitis B virus infection is a disease that is associated with significant morbidity
and mortality. Hepatitis D virus infection, which requires a coinfection with
hepatitis B, is associated with an increased morbidity and mortality over that of
hepatitis B virus infection alone. It is transmitted during sexual intercourse through
contaminated body fluids, saliva, and blood; in utero; and at delivery. Lateral
transmission, which is more common than vertical transmission, should be of
particular concern. This virus has the capability of resulting in chronic active
infection, cirrhosis, liver failure, and ultimately death.
One method of preventing the spread of this serious infection and maintaining
wellness is the hepatitis B vaccine. The recombinant form causes no significant
adverse reactions in the recipient. It is safe to administer, even in pregnancy.
Perhaps a program of vaccinations should be instituted for all individuals who
have yet to be exposed to the hepatitis B virus. A vaccination program would be
particularly prudent for adolescents who are sexually active because these young
people are likely to have multiple sexual partners, therefore raising their risk.
Since the acquisition of this virus may come from a variety of sources, the
vaccination of all pregnant patients who have not been exposed to the virus would
be warranted, especially considering that vaccination in the pregnant patient would
also provide protection for her fetus.
Opinions on this subject would be welcomed from our readers.
Sebastian Faro, M.D., Ph.D.
Editor-in-Chief
Editorial
Received October 17, 1994
Accepted October 18, 1994

				

